# Outbreaks of SARS-CoV-2 Infections in Nursing Homes during Periods of Delta and Omicron Predominance, United States, July 2021–March 2022

**DOI:** 10.3201/eid2904.221605

**Published:** 2023-04

**Authors:** W. Wyatt Wilson, Amelia A. Keaton, Lucas G. Ochoa, Kelly M. Hatfield, Paige Gable, Kelly A. Walblay, Richard A. Teran, Meghan Shea, Urooj Khan, Ginger Stringer, Meenalochani Ganesan, Jordan Gilbert, Joanne G. Colletti, Erin M. Grogan, Carly Calabrese, Andrew Hennenfent, Rebecca Perlmutter, Katherine A. Janiszewski, Christina Brandeburg, Ishrat Kamal-Ahmed, Kyle Strand, Matthew Donahue, M. Salman Ashraf, Emily Berns, Jennifer MacFarquhar, Meghan L. Linder, Dat J. Tran, Patricia Kopp, Rebecca M. Walker, Rebekah Ess, James Baggs, John A. Jernigan, Alex Kallen, Jennifer C. Hunter, Monitoring Outbreaks of Variants in Nursing Homes (MOVIN) Surveillance Team

**Affiliations:** Centers for Disease Control and Prevention, Atlanta, Georgia, USA (W.W. Wilson, A.A. Keaton, L.G. Ochoa, K.M. Hatfield, P. Gable, R.A. Teran, J. MacFarquhar, J. Baggs, J.A. Jernigan, A. Kallen, J.C. Hunter);; Chicago Department of Public Health, Chicago, Illinois, USA (K.A. Walblay, R.A. Teran);; Colorado Department of Public Health and Environment, Denver, Colorado, USA (M. Shea, U. Khan, G. Stringer, M. Ganesan, J. Gilbert);; Connecticut Department of Public Health, Hartford, Connecticut, USA (J.G. Colletti, E.M. Grogan);; Iowa Department of Health and Human Services, Des Moines, Iowa, USA (C. Calabrese, A. Hennenfent);; Maryland Department of Health, Baltimore, Maryland, USA (R. Perlmutter);; Massachusetts Department of Public Health, Jamaica Plain, Massachusetts, USA (K.A. Janiszewski, C. Brandeburg);; Nebraska Department of Health and Human Services, Lincoln, Nebraska, USA (I. Kamal-Ahmed, K. Strand, M. Donahue, M.S. Ashraf);; North Carolina Department of Health and Human Services, Raleigh, North Carolina, USA (E. Berns, J. MacFarquhar);; Oregon Health Authority, Portland, Oregon, USA (M.L. Linder, D.J. Tran);; South Carolina Department of Health and Environmental Control, Columbia, South Carolina, USA (P. Kopp, R.M. Walker);; Utah Department of Health, Salt Lake City, Utah, USA (R. Ess).

**Keywords:** COVID-19, coronavirus disease, SARS-CoV-2, severe acute respiratory syndrome coronavirus 2, viruses, respiratory infections, zoonoses, nursing home, outbreak, Delta, Omicron, booster, United States

## Abstract

SARS-CoV-2 infections among vaccinated nursing home residents increased after the Omicron variant emerged. Data on booster dose effectiveness in this population are limited. During July 2021–March 2022, nursing home outbreaks in 11 US jurisdictions involving >3 infections within 14 days among residents who had received at least the primary COVID-19 vaccine(s) were monitored. Among 2,188 nursing homes, 1,247 outbreaks were reported in the periods of Delta (n = 356, 29%), mixed Delta/Omicron (n = 354, 28%), and Omicron (n = 536, 43%) predominance. During the Omicron-predominant period, the risk for infection within 14 days of an outbreak start was lower among boosted residents than among residents who had received the primary vaccine series alone (risk ratio [RR] 0.25, 95% CI 0.19–0.33). Once infected, boosted residents were at lower risk for all-cause hospitalization (RR 0.48, 95% CI 0.40–0.49) and death (RR 0.45, 95% CI 0.34–0.59) than primary vaccine–only residents.

After its detection in November 2021, the SARS-CoV-2 B.1.1.529 (Omicron) variant was labeled a variant of concern by the World Health Organization (*1*). Compared with previous variants, Omicron had at least 37 mutations identified in the spike gene, raising concerns about reduced antibody binding affinity and the potential for immune escape (*2*,*3*). Among the US adult population, studies demonstrated that the odds of symptomatic infection are higher for the Omicron variant than Delta (*4*). Conversely, the odds of a subsequent severe outcome, such as COVID-19 hospitalization and death, have been lower among Omicron infections than Delta infections (*5*).

In the United States, nursing home residents have been disproportionately affected by COVID-19 (*6*,*7*). Most nursing home residents are older adults with chronic comorbidities who live in congregate settings where infection control strategies are difficult to implement but vital to preventing facility transmission (*8*). As a result, nursing home residents were prioritized to receive the primary COVID-19 vaccine series (*9*), as well as vaccine booster doses (*10*,*11*). During September–October 2021, the Centers for Disease Control and Prevention (CDC) recommended use of a single COVID-19 vaccine booster dose for all persons >18 years of age, to be given 6 months after receipt of a primary mRNA vaccination series or 2 months after receipt of a primary Johnson & Johnson/Janssen (https://www.jnj.com) vaccine dose. In January 2022, CDC updated booster recommendations to shorten the interval to 5 months for receiving an mRNA booster after primary mRNA vaccination series (*12*). However, data on SARS-CoV-2 infections and outcomes among nursing home residents during the Omicron-predominant period of the COVID-19 pandemic have been limited. One study from long-term care facilities in England found reduced risk for severe outcomes among SARS-CoV-2–positive residents during the Omicron period compared with the pre-Omicron period (*13*). An analysis of nursing home data from the US National Healthcare Safety Network (NHSN) found that a COVID-19 booster dose provided greater protection against infection (relative vaccine effectiveness = 46.9%) than the primary vaccine series alone during the Omicron period (*14*). However, NHSN data are limited to aggregate reporting at the facility level; few studies have examined resident outcomes within the context of facility outbreaks.

As part of a surveillance effort to monitor SARS-CoV-2 outbreaks in nursing homes (*15*), CDC partnered with a subset of US public health jurisdictions to prospectively monitor COVID-19 outbreaks in nursing homes. We targeted outbreaks beginning July 26, 2021–January 31, 2022, in which >3 SARS-CoV-2 infections occurred within 14 days among residents who had completed at least a primary COVID-19 vaccination series. We describe outbreak characteristics during periods of Delta, mixed Delta/Omicron, and Omicron variant predominance; compare the risk for resident outcomes such as infection, all-cause hospitalization, and all-cause death in the Omicron and Delta periods; and examine risk for those outcomes by booster status during the Omicron period.

## Methods

### Overview of Health Department Recruitment and NH Outbreak Surveillance

We invited CDC-funded healthcare-associated infections programs in health departments to participate in the surveillance project. Participating health departments provided the number of facilities under surveillance and collected outbreak, facility, and resident information from facilities with eligible outbreaks, which were defined as those with >3 SARS-CoV-2 infections within a 14-day period in residents who received at least a primary COVID-19 vaccine series. Outbreaks were identified using data reported to NHSN and other state-based surveillance systems.

Infection in a resident who had received a primary vaccine series alone was defined as a positive SARS-CoV-2 viral nucleic acid amplification or antigen test result collected from a respiratory specimen in a resident who had completed a primary vaccination series (2 doses of the Pfizer-BioNTech [https://www.pfizer.com] or Moderna [https://www.modernatx.com] mRNA vaccine or 1 dose of the Johnson & Johnson/Janssen vaccine) at least 14 days earlier. For this analysis, residents were considered boosted 14 days after receipt of an additional primary series dose or booster dose (*9*). Surveillance data could not distinguish between immunocompromised nursing home residents who received an additional primary vaccine and residents who received a booster dose. As such, the category of residents who were vaccinated with an additional or booster dose and are designated as boosted in this analysis included residents who received 2 primary mRNA doses followed by a booster dose; 3 primary mRNA doses; 3 primary mRNA doses followed by a booster dose (i.e., 4 total doses); 1 primary Janssen dose followed by a booster dose; 1 primary Janssen dose and an additional primary mRNA vaccine dose; or 1 primary Janssen dose and an additional primary mRNA vaccine dose followed by a booster dose (*12*). The surveillance definition of boosted used for this study was defined before CDC released the recommendation for when to consider an individual up to date, which is immediately after getting all recommended boosters (*16*). Staff with a positive SARS-CoV-2 test result were not counted toward the infection criteria for an eligible outbreak.

### Surveillance Data Collection

Health departments reported facility-level resident census stratified by vaccination status at the date of outbreak onset, defined as the date of first SARS-CoV-2–positive specimen collection in a resident or staff member after a period of >14 days with no resident or staff infections. Other outbreak information collected included outbreak onset date, outbreak closure date (defined as 14 days after last identified SARS-CoV-2 infection in a resident or staff member), and whether the initial infection was detected in a resident or staff member. Information collected for infected residents included whether they were in the facility on outbreak onset date, vaccination status at time of SARS-CoV-2–positive specimen collection date, all-cause hospitalization, all-cause death, and SARS-CoV-2 variant type (if known). Hospitalization from any cause was monitored and recorded through date of outbreak closure; death from any cause was monitored and recorded through 14 days after outbreak closure date.

### Surveillance Period

Jurisdictions reported new outbreaks in which onset occurred during July 26, 2021–January 31, 2022, and continued reporting new resident infections until meeting the outbreak closure definition or the end of the infection surveillance period (February 28, 2022). We excluded outbreaks from analyses if reported by health departments that were unable to participate for the entire surveillance period or if outbreak status was listed as unknown at the end of the surveillance period. Any unclosed outbreaks at the end of the infection surveillance period were classified as still active; for these outbreaks, hospitalization and deaths were monitored and recorded for another 14 days and 28 days from the end of the infection surveillance period (March 14 and 28, 2022). Outbreak duration was the number of days from outbreak onset until closure. Because outbreaks with onset on the last day of the outbreak surveillance period (January 31, 2022) had a maximum of 42 days to close (14 days after last resident infection or 14 days after the end of the infection surveillance period on February 28, whichever was earlier) and because a subset of outbreaks continued beyond the infection surveillance period, outbreak duration was categorized into 3 groups: <28 days, 29–41 days, and >42 days.

### Outbreak Characteristics

We analyzed and described outbreak characteristics including outbreak duration and size, initial infection, and SARS-CoV-2 variant identified by whole-genome sequencing (Appendix) by period based on outbreak onset date: Delta (July 26–November 1, 2021), mixed Delta/Omicron (November 2–December 18, 2021), and Omicron (December 19, 2021–January 31, 2022). Periods were defined as the range of dates when the estimated national prevalence for a specific SARS-CoV-2 variant was >75% (*17*). We compared outbreak characteristics across Delta and Omicron periods by using χ^2^ or Wilcoxon rank-sum tests for categorical and nonnormally distributed continuous variables. We did not include the mixed Delta/Omicron period in analytic comparisons.

### Resident Outcomes by Delta and Omicron Period

We used a Poisson generalized estimating equation (GEE) model with log links, adjusting for facility-level clustering, to estimate the risk for infection in the first 28 days of the outbreak for residents with a completed primary COVID-19 vaccine series who were present at outbreak onset in the Delta and Omicron periods. To enable comparison across all outbreaks, we restricted our analysis to the first 28 days because outbreaks with onset during the last day of surveillance had a maximum of 28 days to register infections. In addition to general exclusion criteria, we excluded additional outbreaks if available sequencing data indicated the outbreak variant was discordant with the outbreak period (i.e., Delta variant during the Omicron period), if multiple variants were identified in a single outbreak, or if resident census data were incomplete.

We compared the risk for severe outcomes among infected residents who had received a primary vaccine series alone by outbreak period (Delta or Omicron) using a binomial GEE regression model with log links adjusting for facility level clustering. We excluded boosted residents because of their limited number in the Delta period.

### Resident Outcomes by Booster Status in the Omicron Period

For Omicron period outbreaks, we compared the risk for resident infection within the first 14 days of an outbreak by booster status at the time of outbreak onset (booster dose vs. primary vaccine series alone) using similar Poisson GEE models along with additional outbreak exclusion criteria as previously described. Because the booster status of residents who did not become infected was available only at outbreak onset, this analysis was restricted to the first 14 days of each outbreak to limit the effect of potential changes in booster status among uninfected residents after outbreak onset.

For the subset of Omicron period outbreaks, we compared the risk for severe outcomes among infected residents by booster status using similar binomial GEE models adjusting for facility clustering. We conducted analyses in SAS version 9.4 (SAS Institute Inc., https://www.sas.com) and defined statistical significance as α = 0.05. This activity was reviewed by CDC and was conducted consistent with applicable federal law and CDC policy (45 C.F.R. part 46.102(l)(2), 21 C.F.R. part 56; 42 U.S.C. Sect. 241(d); 5 U.S.C. Sect. 552a; 44 U.S.C. Sect. 3501 et seq).

## Results

### Outbreak Surveillance

**Figure 1:**
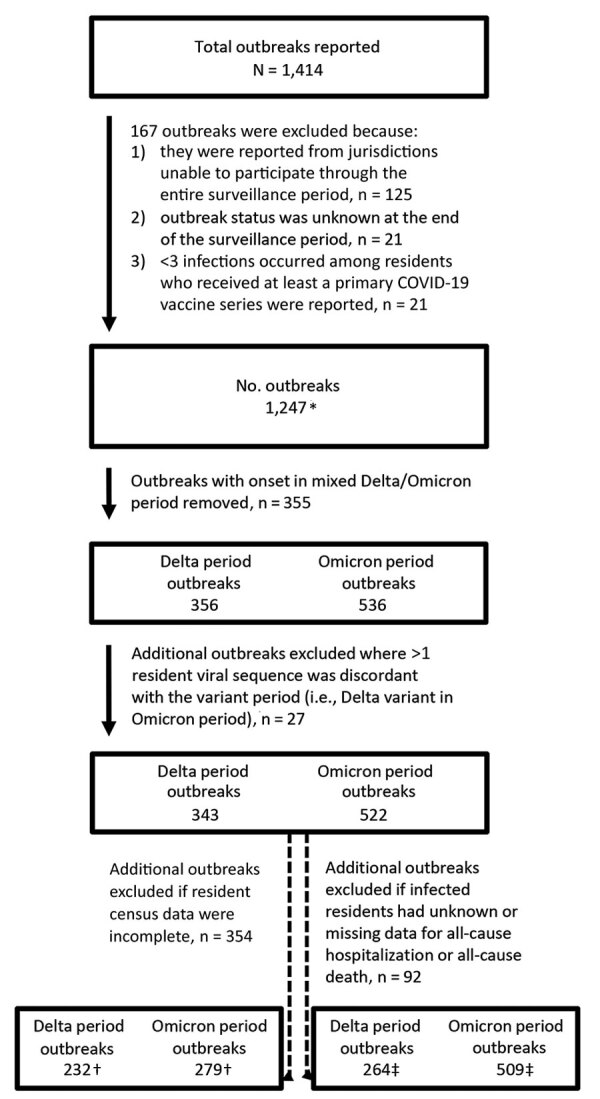
Flow diagram of reported outbreaks included in analysis of SARS-CoV-2 infections in nursing homes during periods of Delta and Omicron variant predominance, United States, July 2021–March 2022. *See Table, Figure 2; †see Figures 3 and 5 for infection attack rate and risk ratio analysis; ‡see Figures 4 and 6 for outcomes attack rate and risk ratio analysis.

During July 26, 2021–January 31, 2022, a total of 1,414 new outbreaks were reported among 16 US jurisdictions (14 state and 2 local health departments). We excluded 167 outbreaks: 125 outbreaks reported by 5 jurisdictions that did not participate for the duration of the surveillance project period, 21 outbreaks with unknown status at the end of the surveillance period (e.g., closed or still active), and 21 outbreaks with <3 infections among residents who had completed at least a primary COVID-19 vaccine series (Figure 1). After excluding outbreaks, we identified 1,247 outbreaks from 11 jurisdictions; the outbreaks occurred in 1,090 (49.8%) of the 2,188 individual facilities represented in the surveillance catchment of the participating US jurisdictions. Overall, the surveillance catchment constituted 84% of the 2,602 licensed nursing homes within participating health department jurisdictions and 14% of the 15,600 nursing homes nationwide (*18*).

### Descriptive Characteristics of Outbreaks

Among the 1,247 outbreaks, 356 (29%) began during the Delta-predominant period of the pandemic (July 26–November 1, 2021), 355 (28%) began during the mixed Delta/Omicron period (November 2–December 18, 2021), and 536 (43%) began during the Omicron-predominant period of the pandemic (December 19, 2021–January 31, 2022) (Table). During the Delta period, viral sequences were reported for >1 infected resident in 53% (n = 189) of outbreaks; the same was true in the Omicron period for 39% (n = 208) of outbreaks. Among outbreaks with a single variant confirmed by sequence data, Delta was the only variant identified among infected residents in 96% (n = 176/183) of outbreaks during the Delta period; similarly, Omicron was the only variant identified among infected residents in 98% (194/198) of outbreaks during the Omicron period. The mixed Delta/Omicron period had the highest proportion of outbreaks in which multiple SARS-CoV-2 variants were identified in infected residents (22%, n = 31/142).

**Table:** Descriptive characteristics of eligible nursing home outbreaks (N = 1,247), by period of SARS-CoV-2 variant predominance, United States, July 2021–January 2022*

Characteristic	Outbreaks beginning during Jul 26–Nov 1: Delta period		Outbreaks beginning during Nov 2–Dec 18: mixed Delta/Omicron period		Outbreaks beginning during Dec 19–Jan 31: Omicron period	
No. outbreaks (% total)	356 (29)		355 (28)		536 (43)	
Individual skilled nursing facilities	347		351		536	
Active outbreaks per week, median (IQR)	146 (100–163)		178 (161–237)		697 (470–838)	
Jurisdiction						
Chicago	8 (2.2)		51 (14.3)		22 (4.1)	
Colorado	63 (17.7)		53 (14.9)		108 (20.1)	
Connecticut	33 (9.3)		38 (10.7)		56 (10.5)	
Iowa	3 (0.8)		1 (0.3)		1 (0.2)	
Maryland	61 (17.1)		92 (25.8)		35 (6.5)	
Massachusetts	40 (11.2)		71 (19.9)		184 (34.4)	
Nebraska	18 (5.1)		2 (0.6)		2 (0.4)	
North Carolina	38 (10.7)		15 (4.2)		21 (3.9)	
Oregon	36 (10.1)		10 (2.8)		34 (6.4)	
South Carolina	34 (9.6)		12 (3.4)		30 (5.6)	
Utah	22 (6.2)		10 (2.8)		43 (8.0)	
SARS-CoV-2 outbreak variant identified among >1 resident						
Delta, B.1.672 and AY.1-AY.107 lineages	176 (49.4)		60 (16.9)		4 (0.7)	
Omicron, B.1.1.529, BA.1, BA.2 lineages	4 (1.1)		50 (14.0)		194 (36.3)	
Alpha	1 (0.3)		0		0	
Other	2 (0.6)		1 (0.3)		0	
Multiple variants identified	6 (1.7)		31 (8.7)		10 (1.9)	
No sequencing information available	167 (46.9)		213 (59.8)		328 (61.3)	
Initial infection in the outbreak						
Staff	220 (61.8)		226 (63.5)		391 (73.1)	
Resident	110 (30.9)		100 (28.1)		77 (14.4)	
Both staff and resident infections at onset	26 (7.3)		29 (8.2)		67 (12.5)	
Unknown or missing	0		0		1 (0.2)	
Days from initial infection to subsequent resident infection in index cluster, median (IQR)	4 (0–11)		5 (0–16)		4 (0–9)	
Median resident census at outbreak onset per outbreak†						
Total no. residents (IQR)	89 (59–114)		98 (74–129)		79 (54–106)	
Residents with booster dose % (IQR)‡	0.0 (0–0)		55.7 (28–75)		74.0 (56–86)	
Residents with primary vaccine series alone, % (IQR)	90.0 (81–96)		38.0 (21–79)		21.0 (11–35)	
Residents partially vaccinated, % (IQR)	1.0 (0–3)		1.0 (0–2.3)		0.0 (0–1.8)	
Residents unvaccinated, % (IQR)	7.0 (3–13)		5.5 (3–11)		5.0 (2–9)	
Median resident infections in first 28 d per outbreak§						
No. infected residents (IQR)	4 (0–10)		6 (1–14)		10 (4–20)	
Infected residents with booster dose, % (IQR)‡	0.0 (0–0)		0.0 (0–18)		35.7 (11–59)	
Infected residents with primary vaccine series alone, % (IQR)	87.5 (71–100)		75.0 (46–100)		46.2 (27–67)	
Infected residents without vaccination, % (IQR)	5.3 (0–25)		8.7 (0–23)		5.0 (0–17)	
Outbreak duration						
<28 d	85 (23.9)		26 (7.3)		16 (3.0)	
29–41 d	93 (26.1)		23 (6.5)		71 (13.3)	
>42 d	178 (50.0)		306 (86.2)		449 (83.8)	
Outbreak status at end of infection surveillance period¶						
Closed	340 (95.5)		308 (86.8)		474 (88.4)	
Still active	16 (4.5)		47 (13.2)		62 (11.6)	

*Values are no. (%) except as indicated. Dates of variant predominance were July 26–November 1, 2021 (Delta), November 2–December 18, 2021 (mixed Delta/Omicron), and December 19, 2021–January 31, 2022 (Omicron). †Resident census data were incomplete for 114 (32%) (Delta period), 133 (37%) (mixed Delta/Omicron period), and 247 (46%) (Omicron period) outbreaks. As a result, denominators used for median resident census at outbreak onset were 242 (Delta), 222 (mixed Delta/Omicron), and 289 (Omicron). ‡Residents who received a booster dose could not be distinguished from residents who received additional vaccine dose. §Infection attack rates were restricted to resident infections present at outbreak onset and those infected within first 28 days of outbreak; all outbreaks included in this analysis had at least 28 days for case ascertainment. ¶All outbreaks included in analysis began before February 1, 2022. Resident infections were reported through February 28, 2022, making March 14, 2022, the last day for an outbreak to close (2 weeks with no infections among residents or staff).

Median time from initial infection in a staff or resident to next identified resident infection (positive specimen collection date) was 4 days (interquartile range [IQR] 0–11 days) and did not differ between the Delta and Omicron periods. Among closed outbreaks (95.5% in Delta period, 88.4% in Omicron period), 67% of infections occurred during the first 28 days. The median number of residents present at outbreak onset and infected in the first 28 days in Omicron period outbreaks (10, IQR 4–20) was greater than in Delta period outbreaks (4, IQR 0–10) (p<0.001). Half (n = 178) of all outbreaks beginning in the Delta period lasted >42 days, compared with 84% (n = 449) during the Omicron period (p<0.001).

### New and Active Outbreaks by Calendar Week

**Figure 2:**
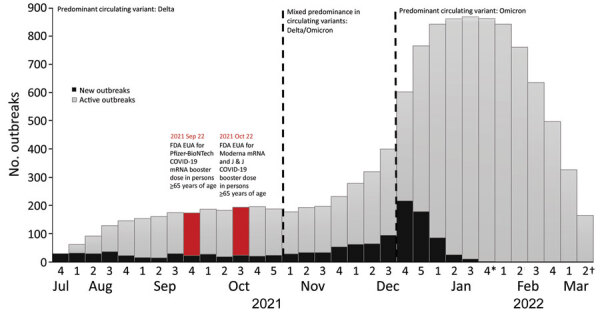
SARS-CoV-2 outbreaks in nursing homes (n = 1,247) by calendar time in weeks, United States, July 2021–March 2022. *January 31, 2022 was the last date for reporting new outbreaks; †resident infections for active outbreaks were reported through February 28, 2022, making March 14, 2022 the last date for an outbreak to close. Outbreaks that were not reported as closed by that date were considered still active. EUA, Emergency Use Authorization; FDA, Food and Drug Administration; J&J, Janssen/Johnson & Johnson, https://www.jnj.com; Pfizer-BioNTech, https://www.pfizer.com.

The number of new outbreaks per week was highest during the fourth week of December 2021 (n = 217) and lowest during the last week of January 2022 (n = 2), after which surveillance reporting of new outbreaks stopped (Figure 2). The number of active outbreaks per week in the Omicron period peaked in the fourth week of January 2022 (n = 861) and was 5 times greater than the peak of active outbreaks per week in the Delta period (n = 175), which occurred in the fourth week of October 2021.

### Resident Attack Rates and Risk Ratios for Outcomes in Residents Who Received a Primary Vaccine Series Alone by Delta and Omicron Periods

**Figure 3:**
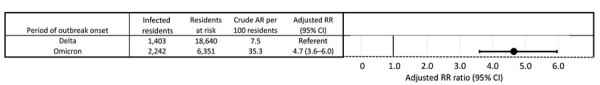
SARS-CoV-2 infection ARs and RRs per 100 nursing home residents by periods of Delta (n = 232) and Omicron (n = 279) variant predominance, United States, July 2021–March 2022. Values are given for residents who had received a primary vaccine series alone, adjusted for facility-level clustering. Resident infections were restricted to the first 28 days of outbreak because all reported outbreaks had at least a 28-day period for case ascertainment. AR, attack rate; RR, risk ratio.

**Figure 4:**
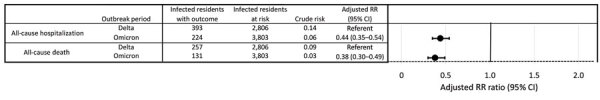
Crude risk and RRs for all-cause hospitalization and all-cause death among SARS-CoV-2–positive nursing home residents who had received a primary COVID-19 vaccine series alone by periods of Delta (n = 264) and Omicron (n = 459) variant predominance, United States, July 2021–March 2022. Values are adjusted for facility-level clustering. RR, risk ratio.

Among residents who had received the primary vaccine series alone, the estimated risk for infection in the first 28 days of an outbreak was significantly higher during the Omicron period (35.0 [95% CI 29.3–40.1]/100 residents) than during the Delta period (7.5 [95% CI 6.2–9.0]/100 residents,) (risk ratio [RR] 4.7, 95% CI 3.6–6.9; p<0.001) (Figure 3). However, the estimated risk for severe outcomes was less than half for infected residents who had received a primary vaccine series alone during the Omicron period, including all-cause hospitalization (RR 0.44, 95% CI 0.35–0.54) and all-cause death (RR 0.38, 95% CI 0.30–0.49), compared with infected residents who had received a primary vaccine series alone during Delta period outbreaks (Figure 4).

### Resident Attack Rates and Risk Ratios for Outcomes in the Omicron Period by Booster Status

**Figure 5:**
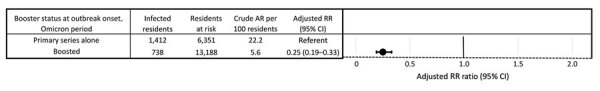
SARS-CoV-2 infection AR and RR per 100 nursing home residents by booster status at outbreak onset during Omicron period outbreaks alone (n = 279), United States, July 2021–March 2022. Values are adjusted for facility-level clustering. AR, attack rate; RR, risk ratio.

**Figure 6:**
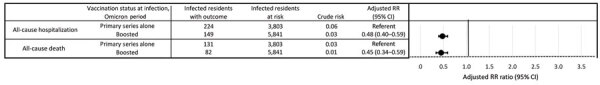
Crude risk and RR for all-cause hospitalization and all-cause death for SARS-CoV-2–positive nursing home residents by vaccination status at time of infection among Omicron period outbreaks alone (n = 509), United States, July 2021–March 2022. Values are adjusted for facility-level clustering. RR, risk ratio.

The estimated risk for infection during the first 14 days of an Omicron-period outbreak was significantly lower among boosted residents (5.6 [95% CI 4.6–6.7]/100 residents) than among residents who had received a primary vaccine series alone (22.2 [95% CI 18.2–26.7]/100 residents) (RR 0.25, 95% CI 0.19–0.33; p<0.001) (Figure 5). Infected boosted residents were half as likely to have all-cause hospitalization (RR 0.48, 95% CI 0.40–0.59) and all-cause death (RR 0.45, 95% CI 0.34–0.59) than were residents who had received a primary vaccine series alone (Figure 6).

## Discussion

By focusing on SARS-CoV-2 outbreaks in nursing homes during the Delta and Omicron periods, this study enables us to describe the effect of these variants within affected facilities and among residents. Five times as many active outbreaks occurred in nursing homes during the peak week of the Omicron period as occurred in the peak of the Delta period. In addition, outbreaks during the Omicron period were significantly larger and longer, and a greater percentage of residents were infected within the first 4 weeks of an outbreak. In contrast, once infected, residents vaccinated with a primary series alone had half the risk of being hospitalized or dying from any cause during the Omicron period than residents vaccinated with a primary series alone in the Delta period. During the Omicron period specifically, 66% (26,992/40,782) of residents had received a booster dose at the time of outbreak onset. Boosted residents in this period were 4 times less likely to get infected in the first 14 days of the outbreak than residents who had received a primary vaccine series alone and, among those infected, they were half as likely to be hospitalized or die from any cause during the follow-up period. In summary, the Omicron period was characterized by an abrupt rise in nursing home outbreak activity, with longer lasting and larger outbreaks compared with the Delta period; fortunately, some of these effects were offset by lower rates of resident hospitalization and death, particularly among persons who received a vaccine booster.

Reported outbreaks in nursing homes increased in December 2021, coinciding with the emergence of the Omicron variant in the United States (*19*). The number of active outbreaks increased over subsequent weeks, mirroring community rates, reflecting the enhanced transmissibility of the Omicron variant and reduced vaccine effectiveness compared with other variants (*4*,*14*). This rise also coincided with worsening staff shortages in nursing homes and increased visitation during the winter holiday season after COVID-19 visitor restrictions were relaxed (*20*). Our data reinforce that persons infected with the Omicron variant are less likely to suffer severe outcomes than are persons with Delta variant infections, both in the general population (*21*) and in nursing homes (*13*). Despite all-cause hospitalization rates being lower during Omicron relative to Delta, the crude number of total hospitalizations among infected residents who had received a booster or primary series during the Omicron period (n = 373) was similar to the number of hospitalizations among infected residents who had received a primary vaccine series alone in the Delta period (n = 393) and, moreover, occurred over a shorter period. Although we were unable to ascertain whether resident hospitalizations resulted from their SARS-CoV-2 infection, our findings indicate that both Delta and Omicron nursing home outbreaks likely placed substantial strains on the US healthcare system.

An analysis of aggregated weekly data from US national surveillance of skilled nursing facilities during February 14–March 27, 2022 (when Omicron was the predominant strain), found that boosted residents were half as likely to be infected (attack rate 2.6, 95% CI 2.5–2.7) than residents with a primary series alone (attack rate 5.0, 95% CI 4.9–5.1) (*14*). Our findings demonstrated similar but greater protection against infection after a booster dose, which was likely caused by several factors, such as a different surveillance period, population, and methodologic approach. Our analysis surveyed outbreaks earlier in the Omicron period, involved a subset of all facilities reporting to NHSN, and used resident-level data (rather than aggregate weekly reporting) to calculate attack rates.

The first limitation of this study is that, without the ability to link US public health and medical records at a national level, we relied on voluntary participation from health departments, which required time-intensive active follow up with facilities and laboratories. As a result, data collection required balancing the burden placed on health departments and nursing home staff with study goals. To ensure collection activities were tenable, we limited reporting of resident census and vaccination status of all residents to one time point (outbreak onset) and did not track resident movements (including discharges, transfers, and admissions). Second, surveillance definitions and recommendations for testing (*22*) were standardized, but investigation practices might have varied by jurisdiction depending on available resources, local policy, and nursing home availability or willingness to share data. That variability could have led to differences in case and outbreak ascertainment, classification of vaccination status at outbreak onset, and verification of hospitalization and death information for cases. Further, it was not feasible to ascertain whether SARS-CoV-2 was the primary reason for hospitalization or cause of death among infected residents. Fourth, the outcome follow-up period was linked to outbreak end date, which led to variations in follow-up times for individual infected residents. Adjusted estimates were not able to account for potential differences in time from infection to outcome by vaccination status or outcomes that occurred beyond the follow-up period. Fifth, specimens with sequence results were only available in ≈50% of all outbreaks because of varying protocols for specimen collection and retention. A sensitivity analysis of attack rates for severe outcomes was restricted to infected residents with confirmed sequencing and demonstrated similar results (Appendix Figure). Sixth, outbreak duration and total infected resident count were likely underestimated in outbreaks that were still active at the end of infection surveillance. As a result, we limited our analysis of outbreak duration and infection attack rates to the minimum number of days collected for all outbreaks.

Residents who received a booster dose might have differed from residents who received a primary vaccine series alone in ways that were not measured, such as medical history, previous SARS-CoV-2 infection and subsequent infection-induced immunity, end-of-life care, or even residing at a facility with enhanced infection prevention practices. In addition, the vaccination status of residents who were not SARS-CoV-2 positive was only reported at outbreak onset. To minimize the effect of unmeasured change in booster status during the course of the outbreak, we focused our comparison of infection attack rates by booster status recorded at outbreak onset and limited it to infections occurring during the first 14 days of the outbreak. Because of those limitations, we did not calculate formal vaccine effectiveness estimates.

Efforts to ensure residents stay up to date with COVID-19 vaccination schedules, which includes additional and booster doses (*23*), are critical to preventing SARS-CoV-2 infection and severe outcomes in nursing home outbreaks. Additional emphasis should be placed on vaccination programs alongside recommended infection prevention and control strategies for long-term care facilities during SARS-CoV-2 outbreaks (*22*), which have the potential to overburden existing nursing home capacities. Continued surveillance of nursing home outbreaks and associated infection attack rates and severe outcomes is warranted as new SARS-CoV-2 variants emerge.

Appendix Additional information about outbreaks of SARS-CoV-2 infections in nursing homes during periods of Delta and Omicron predominance, United States, July 2021–March 2022
