# Supplementary material for: Outbreaks of SARS-CoV-2 Infections in Nursing Homes during Periods of Delta and Omicron Predominance, United States, July 2021–March 2022
Source: Emerg Infect Dis. 2023 Apr;29(4):761–70. doi: 10.3201/eid2904.221605 (PMC10045674; doi:10.3201/eid2904.221605)
Supplement: Appendix — Additional information about outbreaks of SARS-CoV-2 infections in nursing homes during periods of Delta and Omicron predominance, United States, July 2021–March 2022 [file 22-1605-Techapp-s1.pdf]

Article DOI: https://doi.org/10.3201/eid2904.221605

EID cannot ensure accessibility for supplementary materials supplied by authors. Readers who have difficulty accessing supplementary content should contact the authors for assistance.

Outbreaks of SARS-CoV-2 Infections in Nursing Homes during Periods of Delta and Omicron Predominance, United States, July 2021–March 2022

Appendix

Outbreak Variant Determination

When respiratory specimens from infected residents were available, jurisdictions attempted to obtain whole genome sequencing to determine the SARS-CoV-2 variant. When a single variant was identified among one or more infected residents of an outbreak, that variant was designated as the outbreak variant regardless of the number of infected residents without available sequencing data. When more than one variant was identified in an outbreak, the outbreak was categorized as having multiple outbreak variants. When no isolates were available for sequencing, the outbreak variant was classified as unknown. Outbreaks related to multiple variants were excluded from analytic modeling.

A

	Outbreak variant	Infected residents with outcome	Infected residents at risk	Crude Risk	Adjusted RR (95% CI)
All-cause hospitalization	Delta	197	1,449	0.14	REF
	Omicron	55	1,496	0.04	0.29 (0.20-0.41)
All-cause mortality	Delta	154	1,449	0.11	REF
	Omicron	37	1,496	0.02	0.23 (0.15-0.36)

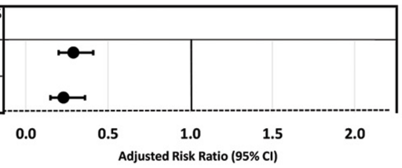

B

	Vaccination status at infection: Omicron outbreaks	Infected residents with outcome	Infected residents at risk	Crude Risk	Adjusted RR (95% CI)
All-cause hospitalization	Primary Series Alone [†]	55	1,496	0.04	REF
	Boosted [‡]	38	2,445	0.02	0.47 (0.30-0.75)
All-cause mortality	Primary Series Alone [†]	37	1,496	0.02	REF
	Boosted [‡]	22	2,445	0.01	0.40 (0.22-0.72)

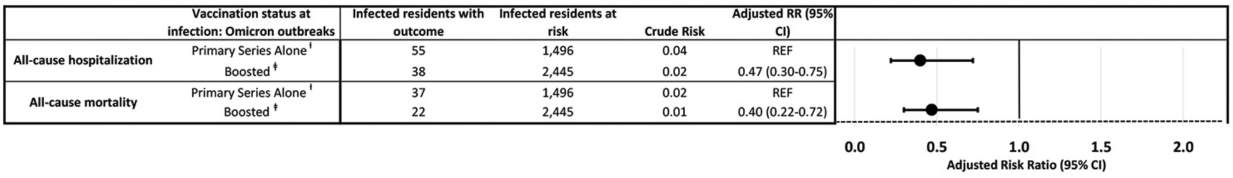

Appendix Figure. A. Crude risk and risk ratios adjusted for facility-level clustering for all-cause hospitalization and all-cause mortality by Delta (n = 131 outbreaks) versus Omicron (n = 169 outbreaks)* period infected residents with a primary COVID-19 vaccine series alone with specimens with Delta-versus Omicron-confirmed sequencing. **B.** Crude risk and risk ratios adjusted for facility-level clustering for all-cause hospitalization and all-cause by vaccination status at time of infection with Omicron-

confirmed sequencing in Omicron outbreaks alone (n = 187). * When analyzing AR and RR of severe outcomes among infected residents with a primary COVID-19 vaccines series alone by Delta and Omicron period outbreaks, 18 outbreaks did not have any infected residents with a primary COVID-19 vaccine series alone and did not contribute data to these analyses. [†] Residents with a primary series vaccination alone refers to any resident who received a primary COVID-19 vaccine series alone (without an additional or booster dose) at time of infection. [‡] Boosted refers to any infected resident who received a primary COVID-19 vaccine series and an additional vaccine or booster dose at time of infection.
